# Effect of extended morning fasting upon *ad libitum* lunch intake and associated metabolic and hormonal responses in obese adults

**DOI:** 10.1038/ijo.2015.154

**Published:** 2015-09-08

**Authors:** E A Chowdhury, J D Richardson, K Tsintzas, D Thompson, J A Betts

**Affiliations:** 1 Department for Health, University of Bath, Bath, UK; 2 School of Life Sciences, Queen's Medical Centre, University of Nottingham, Nottingham, England

## Abstract

**Background/Objectives:**

Breakfast omission is positively associated with obesity and increased risk of disease. However, little is known about the acute effects of extended morning fasting upon subsequent energy intake and associated metabolic/regulatory factors in obese adults.

**Subjects/Methods:**

In a randomised cross-over design, 24 obese men (*n*=8) and women (*n*=16) extended their overnight fast by omitting breakfast consumption or ingesting a typical carbohydrate-rich breakfast of 2183±393 kJ (521±94 kcal), before an *ad libitum* pasta lunch 3 h later. Blood samples were obtained throughout the day until 3 h post lunch and analysed for hormones implicated in appetite regulation, along with metabolic outcomes and subjective appetite measures.

**Results:**

Lunch intake was unaffected by extended morning fasting (difference=218 kJ, 95% confidence interval −54 kJ, 490 kJ; *P*=0.1) resulting in lower total intake in the fasting trial (difference=−1964 kJ, 95% confidence interval −1645 kJ, −2281 kJ; *P*<0.01). Systemic concentrations of peptide tyrosine–tyrosine and leptin were lower during the afternoon following morning fasting (*P*⩽0.06). Plasma-acylated ghrelin concentrations were also lower following the *ad libitum* lunch in the fasting trial (*P*<0.05) but this effect was not apparent for total ghrelin (*P*⩾0.1). Serum insulin concentrations were greater throughout the afternoon in the fasting trial (*P*=0.05), with plasma glucose also greater 1 h after lunch (*P*<0.01). Extended morning fasting did not result in greater appetite ratings after lunch, with some tendency for lower appetite 3 h post lunch (*P*=0.09).

**Conclusions:**

We demonstrate for the first time that, in obese adults, extended morning fasting does not cause compensatory intake during an *ad libitum* lunch nor does it increase appetite during the afternoon. Morning fasting reduced satiety hormone responses to a subsequent lunch meal but counterintuitively also reduced concentrations of the appetite-stimulating hormone-acylated ghrelin during the afternoon relative to lunch consumed after breakfast.

## Introduction

Regular breakfast omission is associated with greater risk of obesity,^1, 2^ prospective weight gain,^3^ type 2 diabetes^4, 5^ and coronary heart disease.^6^ Randomised controlled trials have examined whether breakfast consumption relative to extended fasting is causally related to weight change in free-living overweight/obese individuals.^7, 8^ Owing to the limitations of quantifying energy intake under free-living conditions,^9^ various laboratory-based studies have also examined acute energy intake and immediate metabolic responses to differing breakfast compositions/quantities.^10, 11, 12, 13, 14, 15, 16, 17^ However, the comparison of these acute responses in obese adults under laboratory-controlled conditions remain to be examined in relation to the more fundamental comparison between extended morning fasting and breakfast consumption.

Laboratory paradigms allow careful examination of the metabolic consequences of meal omission and can highlight potential mechanisms that cannot be studied in free-living settings. The use of an *ad libitum* lunch meal also allows some insight into the effects of different morning intakes on immediate energetic compensation that set meals do not allow. Some laboratory studies have quantified *ad libitum* energy intake following breakfast omission in lean individuals,^18, 19, 20, 21, 22^ with the majority of studies indicating additional intake at lunch^18, 19, 21, 22^ but less clear evidence for the effects upon intake at subsequent meal/snacking opportunities.^18, 22^ In all but one investigation^20^ where a relatively small (10% of daily energy requirements) breakfast was consumed, subsequent feeding has been insufficient to compensate for the breakfast omitted.^18, 19, 21, 22^ However, there is no evidence pertaining to immediate energetic compensation following breakfast omission in obese individuals. A recent study has examined metabolic and hormonal responses to extended fasting compared with breakfast consumption in overweight individuals.^23^ However, this was in response to a set lunch meal, so immediate energetic compensation was not examined. It is therefore currently unknown the degree to which obese individuals compensate for morning fasting and importantly, the metabolic and hormonal consequences of doing so.

It cannot be assumed that the responses of obese individuals will be the same as lean individuals. Previous studies contrasting obese and lean counterparts have highlighted several relevant differences that might contribute to divergent findings in the two groups. For example, obese individuals have delayed satiation to feeding, with greater energy intake before reaching maximum satiation.^24^ This discrepancy in satiation may partly be influenced by differences in appetite-regulating hormones. It has been reported that peptide tyrosine–tyrosine (PYY; a hormone implicated in satiety^25^) concentrations are lower postprandially in obese as opposed to normal-weight individuals, despite greater intake during an *ad libitum* buffet lunch.^26^ This attenuation of PYY release has been implicated in the reduced satiety induced by meals in obese individuals.^27^ Furthermore, the usual suppression of ghrelin (an appetite-stimulating hormone^28^) in response to feeding in lean individuals is reduced^29^ or potentially even completely abolished^30^ in obese individuals. Although these studies have established differences in hormonal regulation of appetite between lean and obese individuals, little is known about the hormonal responses of obese individuals to feeding after an extended morning fast and a subsequent meal. Owing to the features of hormonal regulation of appetite in obese individuals outlined above, the potential for a prior meal (in this instance a typical breakfast) to reduce subsequent intake might be inhibited but this requires examination.

To this end, the present study examined energy intake, as well as metabolic and hormonal responses to acute morning fasting in obese individuals. It is hypothesised that extended morning fasting will not result in greater *ad libitum* lunch intake than breakfast consumption.

## Participants and methods

### Participants

Twenty-four healthy obese men (*n*=8) and women (*n*=16) aged 25–58 years took part in this study. Written informed consent was obtained from all participants, with the Ethical Approval for the study obtained from the NHS Bristol Research Ethics Committee. The study is registered with Current Controlled Trials (ISRCTN31521726). Participants were firstly deemed eligible to participate if they had a body mass index of ⩾30 kg m^−2^ and then later classified as obese based upon dual energy X-ray absorptiometry-derived fat mass indices of ⩾9 kg m^–2^ (men) and ⩾13 kg m^−2^ (women).^31^ The study was part of a wider programme of work.^32^ The sample size for this study was based on estimates for the programme of work as described previously.^32^ In brief, those estimates were determined by the number of participants required to detect differences in free-living physical activity and energy intake between two independent experimental groups (*n*~14 per group). Therefore, the sample size of this investigation (*n*=24) is sufficient to detect any meaningful responses of the more tightly controlled laboratory-based parameters assessed within participants using the repeated measures cross-over design employed. Participants reported being weight stable (±2% body mass within the past 6 months), followed a standard sleep-wake cycle (for example, no shift workers) and did not anticipate changes in lifestyle (for example, diet/exercise) during the study period. Participants were free from metabolic disorders and were not taking any medications known to affect appetite regulation, with those participants that were pre-menopausal either menstruating regularly, or following their chosen contraceptive method (that is, pill, implant) for >6 months. Pre-menopausal women were tested during days 3–10 of the menstrual cycle to avoid any effect of menstrual cycle phase on appetite. The cohort comprised of a mixture of regular breakfast consumers and non-consumers (classified based on >50 kcal intake within 2 h of waking on ⩾4 days of the week). Characteristics of participants are presented in Table 1.

### Study methodology

In brief, the obese participants recruited for the current investigation undertook a randomised, counterbalanced cross-over study design comprising two laboratory-based-feeding trials as previously described in full for lean participants^21^ and following general methods as described in the protocol for the wider programme of work.^32^

### Protocol for laboratory visits

Prior to their first visit to the laboratory, participants maintained a 48 h food and drink record, which they subsequently replicated prior to their second laboratory visit. All participants were tested entirely separately from one another and remained sedentary in the laboratory throughout. Upon arrival at 08:00±1 h, following an overnight fast of ⩾10 h duration, participants voided and then had body mass measured in light clothing (Seca 873, Vogel and Halke). Resting metabolic rate was obtained according to guidelines for best practice.^33, 34^ A cannula was then placed into an antecubital vein, with a 15 ml baseline sample of blood drawn. Participants then either consumed a breakfast (to be finished within 15 min) or were asked to rest for an equivalent period, with blood drawn at 15 min, 30 min and an hour after the completion of the breakfast/rest period. Blood samples were then drawn at hourly intervals until 3 h post breakfast, when an *ad libitum* lunch was served. The lunch period lasted 30 min with participants left alone during this time. Following lunch, blood samples were obtained at hourly intervals from 1 h after the end of lunch until 3 h after lunch. Throughout the day, participants completed visual analogue scales assessing appetite.

### Meal provision

During the breakfast trial, the breakfast consisted of typical breakfast foods (that is, cereal with milk, toast and orange juice), in quantities providing 0.06 g carbohydrate per kcal of individually measured daily resting metabolic rate (energy content of breakfast ~31% of resting metabolic rate) for each participant and provided 2183±393 kJ (521±94 kcal), as 70% carbohydrate, 17% fat and 13% protein. The *ad libitum* lunch participants consumed consisted of freshly cooked pasta with a tomato-based sauce, which provided 79% carbohydrate, 14% fat and 7% protein. This was provided in a large bowl, with the pasta topped up every 10 min to prevent visual feedback of food consumed and any tendency to finish the provided portion. The lunch meal was consumed alone and participants were played a recorded message asking them to eat until they had satisfied their hunger.

### Expired gas analysis

Douglas bags were employed to obtain expired air samples with samples for resting metabolic rate collected in line with guidance for best practice.^33, 34^ Rates of both oxygen utilisation (V̇O_2_) and carbon dioxide production (V.CO_2_) were used to calculate energy expenditure^35^ corrected for urinary nitrogen excretion:^36^

Energy expenditure=(3.941 × V̇O_2_)+(1.106 × V.CO_2_)+(2.17 × nitrogen excretion).

### Blood sampling and analysis

Blood was sampled via intravenous cannula inserted into veins of the antecubital region of the arm. Blood was collected and stored as serum or plasma using standard methods, apart from samples for analysis of acylated ghrelin, with blood treated to prevent degradation by proteases as described previously.^21^ Total ghrelin (intra-assay coefficient of variation (CV), 4.0%, inter-assay CV, 7.8%) and acylated ghrelin (intra-assay CV, 4.2%, inter-assay CV, 11.3%) (Bertin Pharma, Montigny le Bretonneux, France) and peptide tyrosine–tyrosine (PYY; intra-assay CV, 4.3%, inter-assay CV, 11.1%) assays were conducted using plasma. Leptin (intra-assay CV, 3.4%, inter-assay CV, 6.4%) (R&D Systems Inc, Abingdon, UK) and insulin (intra-assay CV, 4.7%, inter-assay CV, 12.5%) (Mercodia AB, Uppsala, Sweden) assays were conducted using serum. Assays employed were commercially available enzyme-linked immunosorbent assay conducted following manufacturer instructions, with all samples batch analysed upon study completion and samples from each participant assayed on the same plate. Plasma samples were analysed for non-esterified fatty acids (intra-assay CV, <5%, inter-assay CV, <5%), glucose (intra-assay CV, <5%, inter-assay CV, <6%) and urea (intra-assay CV, <5%, inter-assay CV, <3%) using a Daytona automated analyser (Randox Laboratories, Crumlin, NI, USA) according to manufacturer guidelines using commercially available immunoassays (Randox Laboratories).

### Urine collection

Urine was collected in containers with 5 ml of 10% thymol isopropanol used as a preservative. The urine collected during a measurement period was thoroughly mixed, and a 1-ml aliquot abstracted and stored at −80 °C. Urinary urea concentrations for use in calculations to determine urinary nitrogen excretion were established using a commercially available immunoassay as described above for plasma.

### Appetite sensations

Paper visual analogue scales of 100-mm length were employed to assess subjective appetite. These scales were completed pre- and post breakfast, pre- and post lunch and following a 3 h postprandial period after lunch. Participants marked a response to questions assessing desire to eat, hunger, fullness and prospective consumption with anchor phrases on the ends of each of the scales (for example, not at all hungry vs as hungry as I have ever felt). Higher scores are indicative of greater sensations. A composite appetite score^37^ was calculated using the following formula:

(desire to eat+hunger+(100−fullness)+prospective consumption)/4.

### Statistical analysis

For single comparisons of two means (for example, energy intake at lunch), data were verified as normally distributed using a Shapiro–Wilk test and paired *t*-tests were used for parametric data. For comparison of variables measured throughout the day in each condition (for example, appetite hormones), repeated measures analysis of variance (breakfast/fasting × time point) were conducted with the application of Greenhouse–Geisser corrections to intra-individual contrasts for *ɛ*<0.75, and the Huynh–Feldt correction employed for less-severe asphericity.^38^ Significant interactions were explored using multiple *t*-tests to locate differences between trials at specific time-points, with a Holm–Bonferroni stepwise adjustment employed.^39^ Statistical significance was accepted at *P*⩽0.05. Data are presented in text as mean±s.d.; with figures displaying mean with normalised confidence intervals. These confidence intervals show the comparison between the two trials at each time point, removing inter-individual variation due to the fully paired experimental design.^40^ Statistical analyses were conducted using IBM SPSS statistics version 22 (IBM, New York, NY, USA).

## Results

### Energy intake

**Figure 1:**
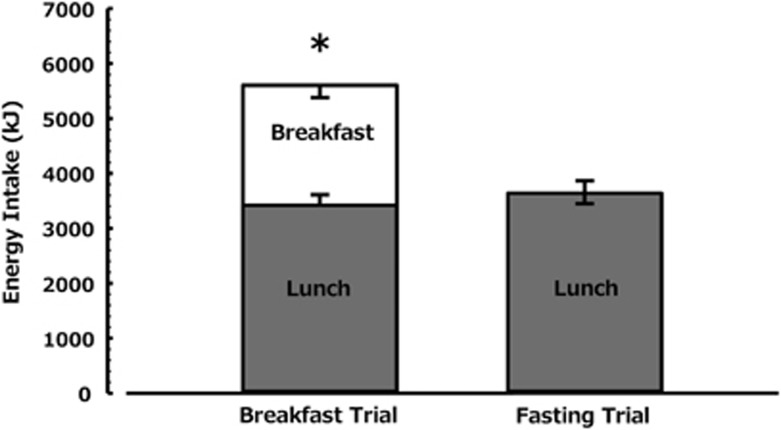
Energy intake during trials. In the morning fasting trial an asymmetric confidence interval is plotted, the negative portion of which reflects the comparison between lunches and the positive portion reflects the comparison against total intake (that is, lunch plus breakfast). An asterisk above a bar represents the comparison between the total intake in the two trials (*n*=24), *P<0.01.

Energy intake at the *ad libitum* lunch was 3638±1480 kJ (869±354 kcal) in the fasting trial and was not significantly different to the breakfast trial (3419±1360 kJ, 817±325 kcal; *P*=0.1, Figure 1). The additional intake at lunch during the fasting trial accounted for 10% of the intake provided with breakfast, resulting in a lower absolute intake over the testing day of 1964 kJ (469 kcal) (95% confidence interval –1645 kJ, –2281 kJ; *P*<0.01) during the fasting trial.

### Glucose

**Figure 2:**
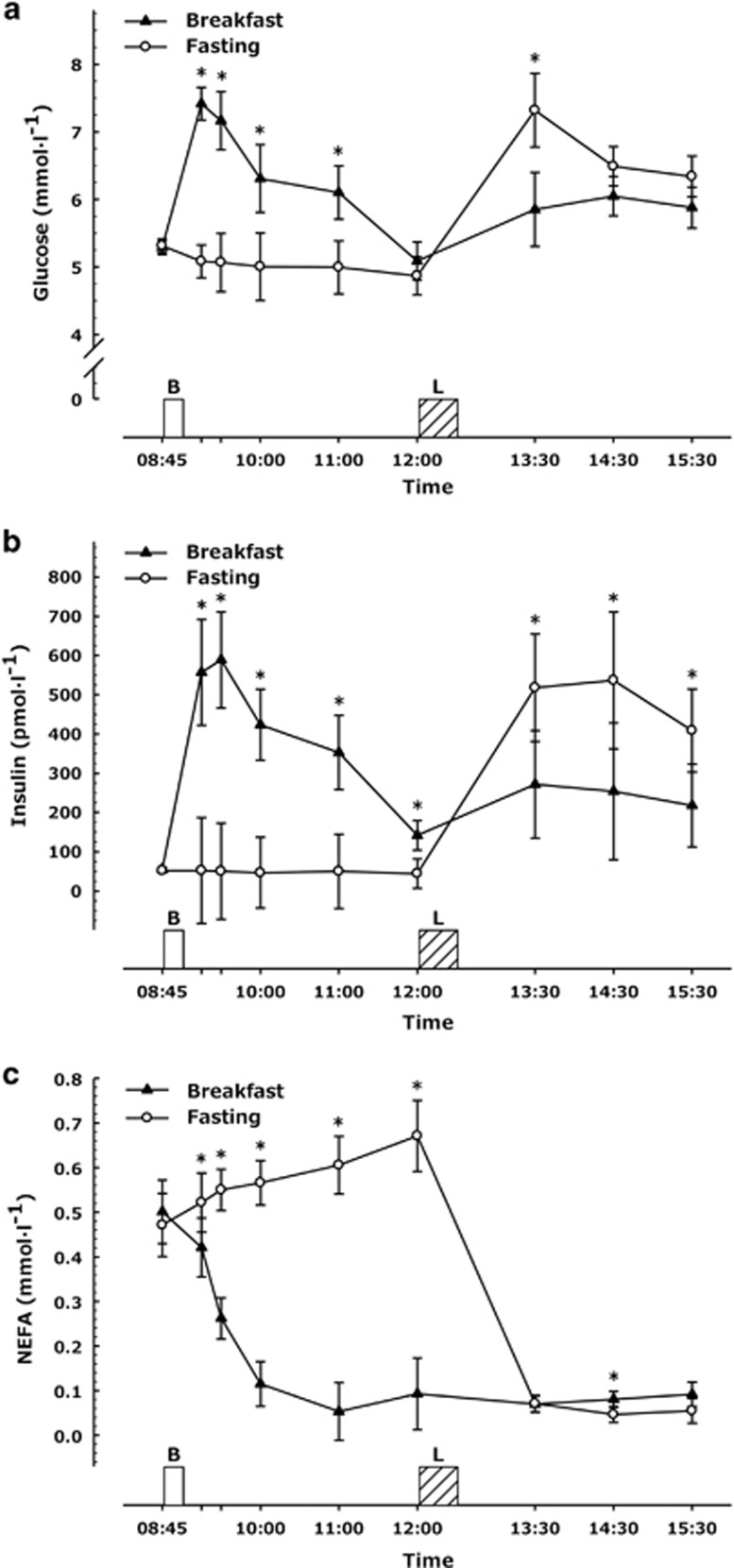
Metabolic responses during trials (**a**) plasma glucose, (**b**) serum insulin, (**c**) plasma NEFA, (all measures *n*=18) where missing data is owing to insufficient blood for analysis. Values represent mean±nCI. *P ⩽0.05 versus corresponding time point in other trial. Annotations on figure represent the following, B=Breakfast period, in which participants ate a prescribed breakfast during the breakfast trial and rested during the morning fasting trial. L=*Ad libitum* pasta lunch.

There were main effects of trial and time, and an interaction of trial × time for plasma glucose (all *P*<0.01). Blood glucose was lower during the fasting trial until 2 h post breakfast (all *P*<0.01; Figure 2a) but was not different 3 h after breakfast (*P*=0.26). Blood glucose concentrations were greater in the fasting trial 1 h after lunch (*P*<0.01) but not throughout the rest of the afternoon (both *P*>0.1).

### Insulin

There were main effects of time, trial and a trial × time interaction for serum insulin concentrations (all *P*<0.02). Insulin concentrations were lower throughout the morning during the fasting trial (all *P*<0.02, Figure 2b). However, after the *ad libitum* lunch, insulin concentrations were greater in the fasting trial (all *P*=0.05).

### Non-esterified fatty acids

Main effects of time and trial were evident for plasma non-esterified fatty acids as well as a time × trial interaction (all *P*<0.01). Non-esterified fatty acids concentrations were higher in the fasting trial throughout the morning (all *P*<0.05, Figure 2c). There was a statistically significant but quantitatively small difference 2 h after lunch consumption (fasting; 0.05±0.02 mmol l^−1^ vs Breakfast; 0.08±0.06 mmol l^−1^; *P*=0.04), with no differences between non-esterified fatty acids concentrations in the two trials at 1 and 3 h after lunch (both *P*>0.1).

### Acylated ghrelin

**Figure 3:**
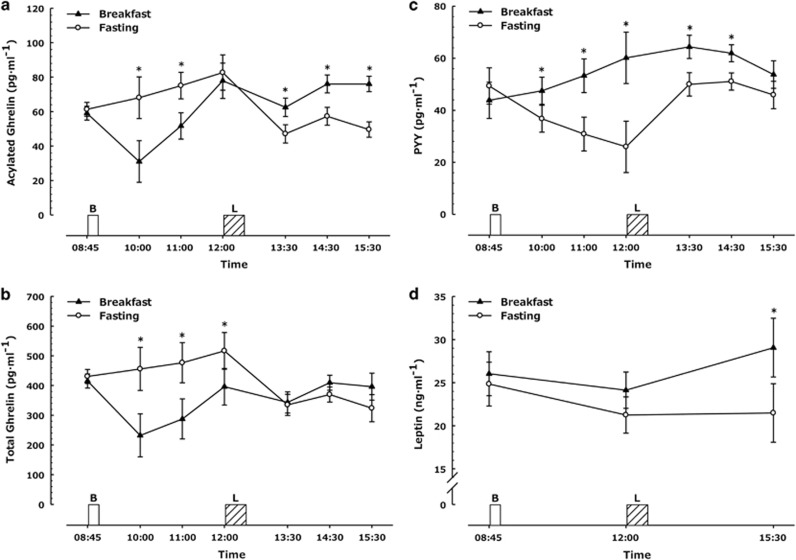
Hormonal responses during trials (**a**) plasma-acylated ghrelin, (**b**) plasma total ghrelin, (**c**) plasma PYY, (**d**) serum leptin, (all measures *n*=18) where missing data is because of insufficient blood for analysis. Values represent mean±nCI. *P<0.05 versus corresponding time point in other trial. Annotations on figure represent the following, B=Breakfast period, in which participants ate a prescribed breakfast during the breakfast trial and rested during the morning fasting trial. L=*Ad libitum* pasta lunch.

A main effect of time and a trial × time interaction (both *P*<0.01) were apparent for plasma-acylated ghrelin concentrations. Acylated ghrelin concentrations were higher in the fasting trial until 2 h post breakfast (both *P*<0.01; Figure 3a), with no difference between the trials immediately prior to the *ad libitum* lunch (*P*=0.47). Following lunch consumption, acylated ghrelin was lower throughout the afternoon in the fasting trial (*P*<0.01).

### Total ghrelin

There were main effects of trial, time and a trial × time interaction (all *P*<0.01) for total ghrelin concentrations. Total ghrelin concentrations were significantly higher in the fasting trial throughout the morning (all *P*<0.04, Figure 3b). Following lunch consumption, there was no difference between total ghrelin concentrations in the two trials.

### PYY

Plasma concentrations of PYY differed over time, between trials and there was a trial × time interaction evident (all *P*<0.01; Figure 3c), with lower concentrations in the fasting trial (all *P*<0.02) until 2 h after lunch. Three hours after lunch consumption, the difference between trials was reduced such that there was only a strong tendency for lower concentrations in the fasting trial (*P*=0.06).

### Leptin

For serum leptin concentrations there were main effects of trial, time and a significant trial × time interaction (all *P*<0.04; Figure 3d). Leptin concentrations decreased pre-lunch in both groups, but with a strong tendency for lower concentrations in the fasting trial (fasting trial, 21.3 ng ml^−1^ vs breakfast trial, 24.1 ng ml^−1^, *P*=0.06). Three hours after lunch consumption, leptin concentrations were significantly lower in the fasting trial (21.5±15.3 ng ml^−1^) than the breakfast consumption trial (29.1±23.0 ng ml^−1^; *P*<0.01).

### Subjective appetite ratings

**Figure 4:**
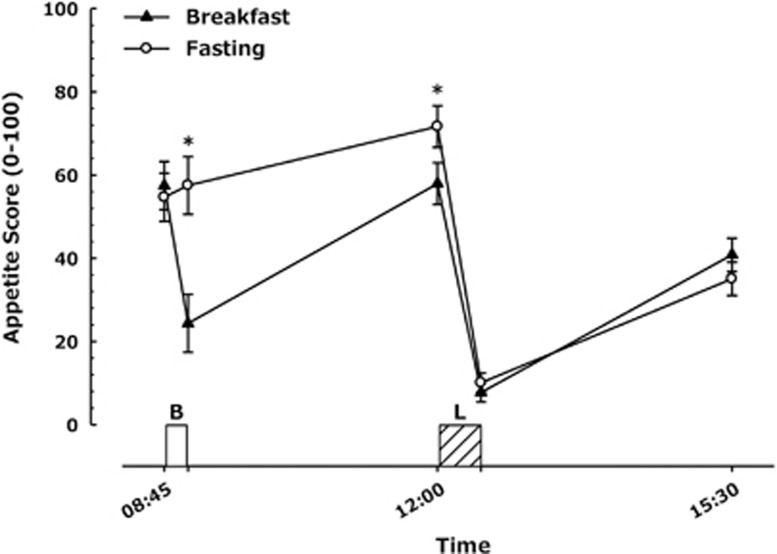
Appetite score during trials. (*n*=24), values represent mean±nCI. *P<0.01 versus corresponding time point in other trial. Annotations on figure represent the following, B=Breakfast period, in which participants ate a prescribed breakfast during the breakfast trial and rested during the morning fasting trial. L=*Ad libitum* pasta lunch.

For the composite appetite score calculated (Figure 4) there was a main effect of trial, time and a trial × time interaction (all *P*<0.03). Appetite was greater in the fasting trial throughout the morning (both *P*<0.01). There was no difference in appetite immediately after lunch consumption (*P*=0.15) but there was a slight tendency for lesser appetite at the end of the testing day in the fasting trial (35±17 vs 41±17; *P*=0.09).

## Discussion

The current study characterised the metabolic, hormonal and appetite responses to extended morning fasting in obese individuals. We have demonstrated for the first time that obese individuals do not compensate for missed breakfast energy intake at a lunchtime meal and that neither ghrelin nor subjective measures of appetite are increased during the afternoon following morning fasting.

This is the first study to specifically examine a contrast of acute morning fasting against breakfast consumption on *ad libitum* lunch intake in obese individuals. Although it has been suggested that energy intake is increased with breakfast skipping^41^ there is accumulating evidence from randomised controlled trials that energy intake is either unaffected^42^ or lower in individuals who skip breakfast.^43, 44^ The current study indicates that any potential compensation for omitted energy intake through breakfast skipping is not strongly manifested through energy intake at this lunchtime meal in obese individuals. This is in contrast to lean individuals previously studied using the same experimental design,^21^ where it was established that energy intake at lunch was significantly increased after morning fasting (although insufficiently to compensate for the omitted breakfast). It therefore appears that increased adiposity results in similar lunch time food intake independent of morning feeding and eradicates the immediate compensatory feeding responses apparent in lean individuals.

It has previously been shown that ghrelin suppression by food intake is either reduced^29^ or abolished^30^ in obese individuals, yet in the present study ghrelin was suppressed by breakfast consumption in that trial, and by lunch intake in the morning fasting trial. This indicates that ghrelin suppression does occur in obese individuals and occurs independent of the overnight fast duration. However, similar to our previous observations in lean individuals,^21^ a prior carbohydrate-rich breakfast meal can interfere with subsequent ghrelin suppression by a similarly carbohydrate-rich *ad libitum* lunch. This blunted response of ghrelin during the afternoon (that is, a lack of suppression after feeding) may be linked to reduced insulin responses to the lunch following breakfast consumption that is putatively due to the second-meal effect^45, 46^ as some authors have proposed an important role for insulin in ghrelin suppression.^47, 48, 49^ Whether this effect persists with breakfast and lunchtime meal combinations inducing less-pronounced differences in insulinaemia relative to morning fasting is an interesting area of future study.

Leptin and PYY concentrations were lower in the afternoon during the fasting trial; as would be expected for both hormones due to the stimulatory effects of repeated feeding^50, 51^ and the proposed mediating role of PYY in signalling relative energy deficit from calorie restriction.^52^ Therefore, in individuals who had fasted during the morning, there were hormonal responses following lunch generally associated with reduced satiety (lower PYY/leptin) as well as others that may contribute to reduced hunger (lower ghrelin). Within the context of these contrasting hormonal responses, there was some tentative evidence of lower perceptions of appetite at the end of the day in the fasting trial in these obese individuals. Future work extending laboratory investigations into the evening would provide interesting information as to the time course of these hormones/subjective perceptions of appetite prior to a 2nd/3rd meal and establish if there are any resultant effects upon total daily energy intake in obese individuals. As it has recently been suggested that the circadian clock increases appetite and hunger in the evening independent of food intake,^53^ it would be informative to establish if different morning feeding patterns can influence appetite later in the day imposed upon this underlying circadian rhythmicity. As two recent publications examining morning fasting in lean individuals have reported insufficient *ad libitum* intake at multiple subsequent meals to compensate for breakfast consumption,^18, 22^ it is of interest to establish the effect of morning fasting upon cumulative intake across several meals in obese individuals.

Despite marked differences in subjective ratings of appetite immediately prior to lunch, there was no difference in lunchtime energy intake between the two trials. This is not completely unexpected as the correlation between pre-lunch appetite ratings and *ad libitum* lunch energy intake in lean men is modest at best (that is, *r*=0.25–0.38)^54^ and a meta-analysis has indicated that sensations of hunger and satiety do not correlate with energy intake in obese individuals.^55^ There are several plausible explanations for this lack of relationship between pre-lunch appetite ratings and energy intake. First, that the relationship between appetite and intake is disrupted in obese individuals.^55^ Second, that subjective appetite measures do not adequately capture the various dimensions of appetite.^56^ Third, there is evidence that people eat according to habit^57, 58^ and it is possible that the current intervention is not potent enough to overcome these feeding habits. Lastly, that another factor led to termination of the meal in participants who were still hungry. This could have arisen owing to feelings other than satiation (that is, boredom and reduced liking for the food provided due to the homogeneity of the meal provided in this fixed course meal), as provision of a variety of foods delays satiation^59^ and promotes greater intake.^60, 61^

Examination of feeding behaviours within the laboratory allows tight experimental control of participants and measurements of physiological parameters that may influence, and are affected by, food intake. However, it is plausible that energy intake in a natural environment would be discrepant from the results obtained in the laboratory owing to self-regulation of feeding patterns and food choices. This is also particularly relevant as obese individuals display elevated neural responses to palatable and energy-dense foods^62^ and are suggested to be more susceptible to environmental cues to eat than hormonal regulation.^63, 64^ Laboratory studies utilising buffet designs or permitting volitional feeding frequency are warranted in obese individuals as well as free-living studies examining energy intake.

The present study demonstrates extended morning fasting does not result in compensatory intake at an *ad libitum* lunch meal in obese individuals. We have shown for the first time that morning fasting does not interfere with the suppression of ghrelin by a lunch in obese individuals, potentially because of the greater insulin response when consuming the meal fasted. There was also no evidence of increased appetite in the afternoon when individuals had fasted throughout the morning. These results indicate that obese individuals do not compensate for morning fasting by consuming more food at the next meal but that hormonal and metabolic responses to lunch are impacted by morning feeding.

## Figures and Tables

**Table 1:** Participant characteristics

*Characteristic*		
*n*		24
Age (years)		44 (10)
Body mass (kg)		96.7 (19.0)
Body mass index (kg m^−^^2^)		33.5 (4.7)
Fat mass index (kg m^−^^2^)^a^	Female	15.1 (3.7)
	Male	9.8 (1.0)
Habitual breakfast consumers (*n*)		14
Female (*n*)		16

a Fat mass index calculated as dual energy X-ray absorptiometry-derived total fat mass divided by height squared. Values represent mean with (s.d.)
